# *Microscopic polyangiitis* With Pauci-Immune Glomerulonephritis Associated With Gastrointestinal Tuberculosis

**DOI:** 10.1155/crin/6619761

**Published:** 2025-02-10

**Authors:** Alexis Monroy-Portillo, Nancy Vargas-San José, Werner De León-Perez, Rodolfo Moreno-Alvarado

**Affiliations:** ^1^Internal Medicine Department, Hospital General San Juan de Dios, Guatemala City, Guatemala; ^2^Renal Pathology Department, SERPAT, Private Pathology Laboratory, Guatemala City, Guatemala; ^3^Nephrology Department, Military Medical Center, Guatemala City, Guatemala

**Keywords:** ANCA, case report, glomerulonephritis, MPA, tuberculosis

## Abstract

**Introduction:** Tuberculosis (TB) is a prevalent disease in Guatemala, present in 20–25 cases per 100 thousand inhabitants. Extrapulmonary TB (EPTB) accounts for only 10%–17% of TB cases. The diagnosis of EPTB is challenging, especially in low-resource settings, because TB can present with clinical characteristics of rheumatological, oncological, or other infectious diseases. Occasionally, mycobacterial infection stimulates the immune system, inducing the generation of antibodies that may lead to autoimmune diseases secondary to primary TB infection, such as vasculitis. To the best of our knowledge, no data have been reported on the prevalence of vasculitis, although some studies worldwide have determined that small-vessel vasculitis is the most common. Here, we present a case report of a male patient with EPTB diagnosed with *Microscopic polyangiitis* (MPA).

**Methods:** A 17-year-old boy with no past medical history visited the emergency room with a three-day history of gastrointestinal bleeding. During hospitalization, acute kidney injury (AKI), disseminated lymphadenopathy, imaging studies, renal biopsy, and immunological tests were performed to confirm the diagnosis.

**Results:** Endoscopy revealed a duodenal lesion containing *Mycobacterium TB* DNA. Further investigation of AKI led to autoimmune serological tests and kidney biopsy, confirming the diagnosis of antineutrophil cytoplasmic antibodies (ANCA)–positive pauci-immune GN. The patient was treated with antituberculous agents, steroids, and plasmapheresis. However, he developed alveolar hemorrhage and respiratory failure leading to death.

**Conclusion:** TB is a common disease in low-income countries, with the pulmonary form being the most common presentation; however, the bacteria can spread to any organ, known as EPTB. It is important to consider that the inflammatory reaction associated with any form of TB can generate other types of noninfectious inflammatory diseases, such as ANCA–positive pauci-immune GN.

## 1. Introduction

TB is a worldwide infectious threat caused by *M. tuberculosis (TB)* and is estimated to infect a quarter of the world's population. TB is a common disease in Guatemala with a prevalence of 20–25 cases per 100,000 inhabitants, representing the fifth-highest incidence of TB in Latin America. The central and northwestern regions had the highest rates of TB [1–3]. According to the World Health Organization, in 2017, Guatemalan registries documented 3505 TB cases, with a mortality rate of 2.2 per 100,000 people [4]. Risk factors such as previous TB treatment, HIV infection, indigenous ethnicity, malnutrition, and lower educational attainment were associated with high mortality in this population [1]. TB could be classified, according to the organ affected, as pulmonary TB or extrapulmonary TB (EPTB). The affected organ could be the primary site of infection, or it could be infected via hematogenous or lymphatic spread of bacteria from the primary organ. The most common form of EPTB is lymphadenitis, which can affect any organ, neck lymphadenitis being the most common presentation. Other common manifestations of EPTB are meningitis, ocular, oral, pleuritis, pericarditis, cutaneous, gastrointestinal, genitourinary, and miliary or disseminated forms [5, 6]. While worldwide, 16% of TB cases are EPTB, in Guatemala, the National Registry confirmed 290 EPTB cases in 2013, representing 10% of all cases [3, 5].

ANCA–associated vasculitis (AAV) is characterized by systemic manifestations associated with necrotizing inflammation of small- and medium-sized blood vessels. The presence of serum autoantibodies against (MPO) or proteinase-3 (PR3) is considered part of the pathogenesis by activating neutrophils which attack small vessels [7]. The clinical manifestation of AAV varies based on the clinicopathologic variants, which can be *Microscopic polyangiitis* (MPA), granulomatosis with polyangiitis (Wegener), granulomatosis with eosinophilic polyangiitis (Churg–Strauss), or vasculitis limited to the kidney [8]. MPA typically affects the kidneys and lungs, but skin, nerves, and gastrointestinal tract involvement could also happen. The symptoms may develop insidiously at the beginning with weight loss, loss of appetite, and fever, and subsequently evolve into pulmorenal syndrome, which is the most severe manifestation. The typical and most frequent cause of glomerular injury in all forms of AVV is crescentic and necrotizing pauci-immune glomerulonephritis (GN). The first-line treatment is corticosteroids and immunosuppressants, and in the case of GN, plasma exchange could be used [8–10]. The epidemiology is challenging to identify because the disease is rare, and it is difficult to get a precise case definition. In Guatemala, small-scale studies have reported that small-sized vessel vasculitis is the most common form, followed by medium-sized vessels. Likewise, other studies have shown that most patients with vasculitis present with associated autoimmune diseases such as systemic lupus erythematosus [9, 11, 12].

The pathological mechanism of AAV is mainly autoimmunity; however, on rare occasions, chronic infections such as TB can be a trigger. The immune reaction caused by *Mycobacterium TB* exposes the contents of the neutrophil granules. Molecular mimicry can lead to activation and release of autoantibodies against neutrophils. However, to the best of our knowledge, no data showing the prevalence or incidence of TB-associated GN have been reported yet [13].

Here, we present a case report of a patient with gastrointestinal bleeding, epigastric pain, and weight loss, accompanied by lymphadenopathy, intermittent fever, and night sweats' symptoms that were related to EPTB in the form of gastrointestinal TB (GITB), which was confirmed using a polymerase chain reaction (PCR). GITB is an uncommon form of EPTB and is challenging to diagnose, particularly in low-resource settings. In addition, our patient presented with acute kidney injury (AKI), proteinuria, and antineutrophil cytoplasmic antibodies (ANCAs) positive for MPO, findings that were related to MPA. This finding is rarely described in the literature, and its identification is important because if not diagnosed early and treated appropriately, the consequences can be fatal for the patient.

## 2. Case Presentation

A 17-year-old male Guatemalan with no medical history presented to the emergency room with a three-day history of gastrointestinal bleeding. He had a history of epigastric pain and unquantified and unintentional weight loss associated with axillary lymphadenopathy, fever without diurnal variation, night sweats, and dry cough in the past 3 months. He reported intermittent use of salbutamol since childhood for the treatment of self-diagnosed asthma.

On admission to the emergency room, blood pressure was 110/60 mmHg, heart rate was 64 beats per minute, respiratory rate was 12 breaths per minute, and temperature 36.6°. The patient presented with generalized pallor; white plaques on the tongue suggestive of oral candidiasis; and cervical, supraclavicular, axillary, and inguinal lymphadenopathies. Rectal examination was positive for gastrointestinal bleeding.

Laboratory tests performed on admission showed evidence of leukocytosis with neutrophilia, anemia, and an elevated erythrocyte sedimentation rate. Elevated serum creatinine and blood urea nitrogen levels were documented, in addition to proteinuria with normal urinalysis. Initial autoimmune tests for antinuclear antibodies using immunofluorescence, C3, C4, and rheumatoid factors were normal (Table 1). Renal ultrasonography revealed normal-sized kidneys with increased echogenicity. Chest radiographs were normal with no pathological infiltrates. Chest computed tomography (CT) scan showed a subpleural nodule measuring 9.7 × 10.3 mm in the left lung. Paranasal sinus CT revealed maxillary and sphenoidal sinusitis. Multiple sputum and PCR chain reaction tests for acid-fast bacilli and *Mycobacterium TB* yielded negative results.

**Figure 1:**
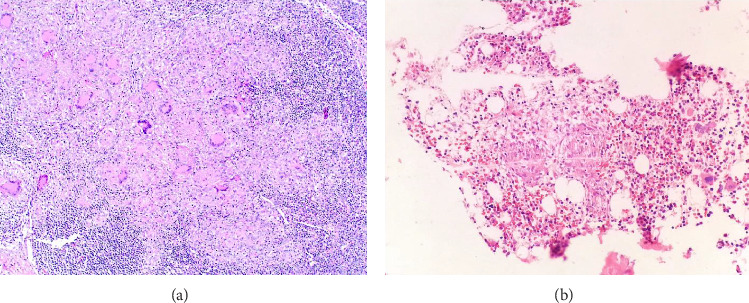
Extracapillary granulomas in inguinal lymphadenopathy and bone biopsy. (a) Inguinal lymphadenopathy pathology in hematoxylin and eosin–stained sections showing granulomatous necrotizing lymphadenitis. (b) Bone biopsy pathology in hematoxylin and eosin–stained sections showing necrotizing granulomas.

Six days after admission, a gastroduodenoscopy revealed an ulcerated duodenal mass and biopsies were performed. Histological analysis performed 17 days later revealed nonspecific inflammation. Inguinal lymph node biopsy revealed necrotizing granulomatous lymphadenitis with no evidence of microorganisms in histological methods or cultures. A bone biopsy showed evidence of a granulomatous process. Granulomas were absent in the blood vessels (Figure 1).

**Figure 2:**
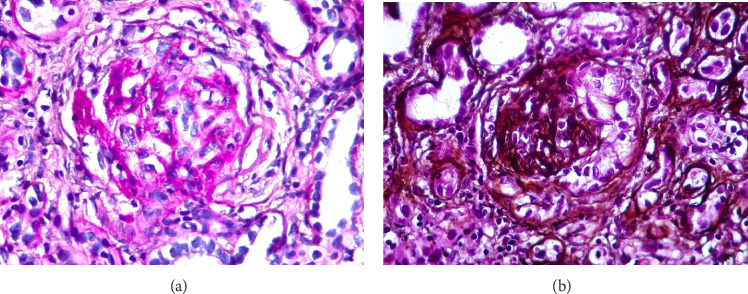
Kidney biopsy showing cellular crescents, endocapillary hypercellularity, and marked retraction of the glomerulus. (a) Glomerulus in hematoxylin and eosin stain with cellular crescents. (b) Glomerulus in periodic acid-Schiff (PAS) with cellular crescents.

Two weeks later, a renal biopsy showed 40 globally sclerosed glomeruli (4/40) and glomerulomegaly (24/40) with extracapillary hypercellularity with crescents and without fibrinoid necrosis. Red cell casts were observed in tubules with mild atrophy. The absence of interstitial fibrosis and negative immunofluorescence were suggestive of pauci-immune GN associated with vasculitis ANCA–positive for MPO (Figure 2). Consequently, steroids and plasmapheresis every 48 h were started.

Due to persistent gastrointestinal bleeding, a second gastroduodenoscopy, performed 25 days after admission, showed an ulcerated lesion in the duodenum, which displayed severe chronic inflammation and granulomatous reaction. PCR for *M. TB* yielded a positive result.

The presence of *M. TB* was not documented in histological or molecular analyses of lymph nodes, bone marrow, bone biopsy, or renal biopsy samples; however, due to positive PCR results for *M. TB* in small bowel tissue, anti-TB treatment (ATT) was started. The patient developed alveolar hemorrhage requiring mechanical ventilation but died after a second episode of alveolar hemorrhage.

## 3. Discussion

GITB is a rare form of EPTB that presents with a variety of symptoms, including abdominal pain, fever, nausea, vomiting, diarrhea, and hemorrhage. Owing to the diversity of symptoms, a high index of clinical suspicion is required to make an accurate diagnosis [14]. In the present case, isolation of the germ in a duodenal biopsy confirmed the diagnosis of GITB, which is a rare manifestation of the disease. In some reports, abdominal TB represented between 12% and 15.8% of EPTB cases in low- and middle-income countries. However, these reports included gastrointestinal, peritoneal, lymph node, and solid viscera. GITB can present with abdominal pain, fever, anorexia, nausea, vomiting, diarrhea, perforations, ulcerations, fistulae, strictures, and hemorrhages, as observed in our patient. Due to its nonspecific symptoms, diagnosis is exceptionally challenging, especially in low-resource settings, as it mimics other diseases. PCR, which was used to determine the diagnosis of TB in our patient, had a sensitivity of 35%–75% and a specificity of 100% [5, 14].

Although there are renal manifestations associated with TB, usually they are reported secondary to direct infection or amyloidosis, but GN is exceptionally rare. In 2024, Forster et al. presented a series of 62 studies that included 130 patients with various types of GN associated with TB, of which approximately 34% of the patients had crescents in renal biopsy (20/59). Among those with pauci-immune GB associated with TB, 100% presented crescents (10/10), of which 30% (3/10) were attributed to drug treatment. Although both AAV and TB share some characteristics that make them difficult to differentiate, vasculitis should be considered in the differential diagnosis of a patient with TB and AKI. The presence of AKI in our patient associated with pulmonary nodules and chronic sinusitis constitutes a clinical indication for ANCA testing [15–17].

To the best of our knowledge, this is the third reported case of ANCA–MPO-–positive pauci-immune GN associated with TB [17]. The underlying mechanisms by which TB may develop vasculitis remain unclear, differing from the known association of AAV and ATT. Several hypotheses on the pathophysiological process by which TB causes kidney damage and GN are as follows: (1) TB modifies humoral immunity, promoting immunocomplex deposition; (2) hypersensitivity reactions to TB wall antigens lead to immunocomplex deposition at the vascular, perivascular, peritubular, and glomerular levels; and (3) in drug-related GN, antibodies against rifampicin have been identified as a potential cause of direct cytotoxic damage to the glomerulus. Isoniazid can also cause systemic necrotizing vasculitis and can be transformed by MPO into active metabolites that develop cytotoxic products. This cytotoxicity is responsible for neutrophil damage and autoantibody production. However, in our patient, since the GN was diagnosed before the initiation of ATT, the medication could not explain the development of the GN. This case emphasizes the rarity and importance of TB and vasculitis and adds to the limited evidence with only two prior documented cases of TB and pauci-immune GN and ANCA positivity [15, 18, 19].

Determination of the exact cause of GN is difficult; however, some specific histopathological characteristics can guide a specific etiology. When granulomas, anti-GMB, amyloidosis, or focal and segmental glomerulosclerosis are identified, they are highly associated with TB itself; however, when biopsy shows minimal changes in disease, it suggests an association with ATT [15, 20].

*Mycobacterium TB* infection causes a chronic inflammatory response, as confirmed by the presence of granulomas in biopsies. Chronic inflammation predisposes the immune system to constantly interact with microbial antigens to generate autoantibodies. TB stimulates the release of neutrophil granule components via interactions with the phenol glycolipids of the bacterial cell wall. The resulting cytokine activation leads to the formation of superantigens that activate white B and T cells. White B cells interact with lysosomal enzymes from neutrophils and develop autoantibodies against the granular components of these cells. Through molecular mimicry, these autoantibodies promote the degranulation of neutrophils, which generates vascular damage typical of vasculitis [13, 19, 21].

Patients with TB could have positive ANCA results with no clinical manifestations of vasculitis, which could be reasonably explained as false positives. In addition, clinical similarities between TB and vasculitis make diagnosis more challenging. However, the pathogenesis of pauci-immune GN involves inflammation of the blood vessels, which was evident on kidney biopsy linked with the positive ANCA test result in our patient. Given the kidney and pulmonary involvement and the histopathological findings, the AAV in our patient was diagnosed as MPA associated with TB. The absence of mycobacteria in kidney samples was helpful in determining that pauci-immune GN could not be explained by the infection itself [19, 22].

Despite potential complications related to immunosuppressive treatment, such as TB and GI hemorrhage, the patient received steroids to treat the manifestations of AAV. The patient was simultaneously treated with plasmapheresis and ATT. The patient's response to ATT could have confirmed the diagnosis of GN secondary to TB, but this could not be achieved because the patient died of alveolar hemorrhage due to advanced chronic disease. Limited resources and accessibility to diagnostic tools are critical factors contributing to delays in diagnosis and treatment, and significantly affecting patient outcomes. In addition, difficulty in accessing high-quality healthcare centers in our country is another factor that affects the prognosis of their condition.

In this case, we considered the diagnosis: TB was confirmed by PCR positive for *M. TB*, and the rapid deterioration of renal function, the histopathological findings of pauci-immune crescentic GN, diffuse alveolar hemorrhage, and positive MPO–ANCA serological tests confirmed the diagnosis of MPA.

## 4. Conclusion

EPTB is a rare disease with a challenging diagnosis, and its spread to the GI is extremely uncommon. MPA with TB is an entity that is rarely reported in the literature. The diagnoses require a high index of suspicion, especially of GITB, due to the absence of specific symptoms. GN should be suspected in the presence of a local or systemic TB infection associated with rapid deterioration of renal function. Early ATT is important for improving prognosis and for considering immunosuppression in the most severe cases of renal damage, such as in the presence of crescents in the renal biopsy or requiring renal replacement therapy. This case report highlights the complex interactions between TB and the development of autoimmune diseases. Recognizing this relationship is important for initiating timely treatment for both entities and improving patient outcomes.

## Figures and Tables

**Table 1:** Laboratory values.

Laboratories		
Variable	Value	Reference range
White blood cells (K/μL)	12.12	4.60–10.20
Neutrophils (K/μL)	10.30	2.0–6.90
Hemoglobin (g/dL)	7.86	12.2–18.10
Erythrocyte sedimentation rate (mm/Hr)	35	< 20
Creatinine (mg/dL)	4.4	0.72–1.25
Urea nitrogen (mg/dL)	50	9.8–20.10
Complement C3 (mg/dL)	105	88–201
Complement C4 (mg/dL)	26	15–45
Rheumatoid factor (IU/mL)	1.5	< 15
HIV test (4th generation)	Negative	Negative
PCR HIV	Non detected	Non detected
24 h urine protein collection (mg/24 horas)	1409	0–300
Protein/creatinine random urine sample (mg/dL)	5.8	< 0.3
Erythrocytes in urine	4 a 5 per field	0–5 per field
Perinuclear antineutrophil cytoplasmic antibodies/myeloperoxidase (pANCA/anti-MPO)	Positive	Negative
Fluorescent antinuclear antibody test (FANA)	Negative	Negative
Cytoplasmic antineutrophil cytoplasmic antibodies/anti-proteinase-3 (cANCA/anti-PR3)	Negative	Negative
Antiglomerular basement membrane antibodies (anti-GBM)	Negative	Negative

## Data Availability

The data that support the findings of this study are available on request from the corresponding author. The data are not publicly available due to privacy or ethical restrictions.
